# The *Enterococcus faecalis* FabT Transcription Factor Regulates Fatty Acid Biosynthesis in Response to Exogeneous Fatty Acids

**DOI:** 10.3389/fmicb.2022.877582

**Published:** 2022-04-25

**Authors:** Qi Zou, Huijuan Dong, Lei Zhu, John E. Cronan

**Affiliations:** ^1^Department of Biochemistry, University of Illinois at Urbana-Champaign, Urbana, IL, United States; ^2^Department of Microbiology, University of Illinois at Urbana-Champaign, Urbana, IL, United States; ^3^College of Life Sciences, Shandong Agricultural University, Taian, China

**Keywords:** phospholipid, transcription, repressor, acyl carrier protein, operons

## Abstract

The phospholipid acyl chains of *Enterococcus faecalis* can be derived either by *de novo* synthesis or by incorporation of exogenous fatty acids through the fatty acid kinase complex (Fak)-phosphate acyltransferase (PlsX) pathway. Exogenous fatty acids suppress fatty acid synthesis through the transcriptional repressor FabT, the loss of which eliminated regulation of *de novo* fatty acid biosynthesis and resulted in decreased incorporation of exogenous unsaturated fatty acids. Purified FabT bound to the promoters of several fatty acid synthesis genes that contain a specific palindromic sequence and binding was enhanced by acylated derivatives of acyl carrier protein B (acyl-AcpB). The loss of the PlsX pathway blocked FabT-dependent transcriptional repression in the presence of oleic acid. Transcriptional repression was partially retained in a *E. faecalis* Δ*acpB* strain which showed decreased fatty acid biosynthesis in the presence of exogenous unsaturated fatty acids. The FabT-dependent activity remaining in the Δ*acpB* strain indicates that acylated derivatives of AcpA were weak enhancers of FabT binding although AcpA is believed to primarily function in *de novo* fatty acid synthesis.

## Introduction

Fatty acid synthesis (FAS) is an almost ubiquitous metabolic pathway that provides precursors for cell membrane bilayer formation, synthesis of secondary metabolites, signaling molecules, and protein post-translational modifications (Beld et al., 2015). In bacteria, mitochondria, and plant plastids the type II fatty acid synthase (FAS II), a set of discrete enzymes, performs this function (Beld et al., 2015). Acyl Carrier Protein (ACP) the carrier of acyl groups during synthesis and utilization, plays an essential role in bacterial fatty acid and phospholipid synthesis (Cronan, 2014; Yao and Rock, 2017). Prior work from this laboratory showed that ACP can also mediate incorporation of exogenous fatty acids (Zhu et al., 2019).

Bacterial FAS II systems are often transcriptionally regulated and regulation was first studied in the gram-negative bacterium *Escherichia coli*, which utilizes FabR and FadR, two TetR superfamily transcription regulators (Schujman and de Mendoza, 2005). In the gram-positive bacterium, *Bacillus subtilis* transcription regulator FapR, a member of the same protein family as the *E. coli* regulators, regulates expression *fab*-related genes that are scattered about the genome (Schujman and de Mendoza, 2005). However, *Streptococcus pneumoniae*, *Lactococcus lactis*, and *Enterococcus faecalis* use the MarR (Multiple Antibiotic Resistance Repressor) family repressor FabT to regulate expression of their clustered *fab* genes (Eckhardt et al., 2013). *S. pneumoniae* FabT has been shown that FabT functions as a dimer to bind palindromic sequences within the promoter regions of related genes using its winged helix-turn-helix (wHTH) domain (Jerga and Rock, 2009). DNA binding by FabT is enhanced by acyl-ACP as the ligand (Jerga and Rock, 2009). The computational model of the *S. pneumoniae* ACP-FabT (Zuo et al., 2019) has been reported and shows the conserved MarR structure. These authors also presented a crystal structure of the interaction of the FabT dimer with the reported DNA binding site. However, as discussed below, the structure seems unreliable since only a single hydrogen bond per monomer is formed between the protein and a DNA base (Zuo et al., 2019).

**FIGURE 1:**
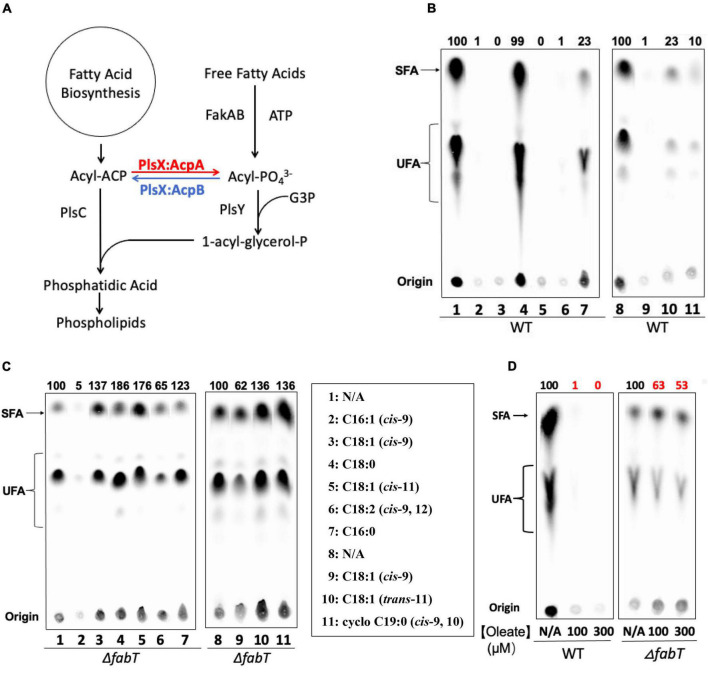
Exogenous fatty acids regulate *E. faecalis de novo* fatty acid biosynthesis by the FabT-transcription factor. **(A)** The *E. faecalis* phospholipid synthesis pathway. **(B)** *De novo* fatty acid biosynthesis in the wild type *E. faecalis* in the presence of exogenous fatty acids. **(C)** *De novo* fatty acid biosynthesis in the *E. faecalis* Δ*fabT* strain in the presence of exogenous fatty acids (100 μM for each fatty acid). **(D)** *De novo* fatty acid biosynthesis of *E. faecalis* wild-type and Δ*fabT* strains in the presence of 100 or 300 μM oleate. For panels **(B,C)**, At the left of lane 1 a lane of cells grown with 10-hydroxyl stearic acid was deleted since this fatty acid is not found in *E. faecalis*. In panel **(D),** three lanes of Δ*acpB* (ZL318) cells between the WT and Δ*fabT* groups were deleted because of the missense mutation later detected in *fabT* gene of this strain (see below).

*Enterococcus faecalis* is an opportunistic pathogen associated with hospital-acquired infections that often show high levels of antibiotic resistance (Fisher and Phillips, 2009). It is a facultative anaerobe that inhabits the gastrointestinal tract of humans and other animals (Fisher and Phillips, 2009; Zhu et al., 2019). Indeed, *E. faecalis* can use either *de novo* synthesized fatty acids or exogenous fatty acids for membrane phospholipid synthesis (Zhu et al., 2013; Zhu et al., 2019; Hays et al., 2021) (Figure 1A). Exogenous fatty acids enter the cells probably by diffusion and are activated by the Fak system which produces acyl-phosphates. The acyl-phosphates can either be directly utilized by the glycerol-3-phosphate acyltransferase PlsY or converted to acyl-ACP by the phosphate acyltransferase PlsX to provide the substrate for the second acyltransferase PlsC to produce phosphatidic acid (Parsons et al., 2015). Atypically *E. faecalis* functionally expresses two ACPs, AcpA and AcpB. AcpA is thought to play the central carrier role in fatty acid synthesis whereas AcpB functions in incorporation of exogenous fatty acids (Zhu et al., 2019) (Figure 1A). *E. faecalis* encodes a FabT that is about 51% identical to *S. pneumoniae* FabT. The *fabT* gene is located at the 5′ end of the *fab* gene operon and FabT transcriptionally regulates expression of both the fatty acid synthesis genes and itself. Deletion of *fabT* strongly increases the proportion of unsaturated fatty acids in cell membrane phospholipids and results in high level of *de novo* fatty acid biosynthesis in the presence of exogenous oleic acid (Zhu et al., 2019).

We report the effects of exogenous fatty acid species on *de novo* fatty acid biosynthesis in *E. faecalis* and demonstrate that FabT is required for this process. Exogenous fatty acids form acyl-AcpB species via the Fak-PlsX pathway. These acyl-AcpB species enhance the ability of FabT to regulate bacterial fatty acid synthesis by repressing transcription of fatty acid synthesis genes located at several chromosomal sites.

## Results

### Exogenous Unsaturated but Not Saturated Fatty Acids Strongly Repress *Enterococcus faecalis de novo* Fatty Acid Biosynthesis

*Enterococcus faecalis* incorporates environmental fatty acids to change its membrane composition (Saito et al., 2018) and our previous work has shown that exogenous oleic acid strongly inhibits *E. faecalis de novo* fatty acid biosynthesis (Zhu et al., 2019). We have now assayed the effects of a variety of fatty acids on *de novo* fatty acid biosynthesis in the wild-type *E. faecalis* FA2-2 strain by [1-^14^C]acetate labeling to test their function compared to oleic acid (Figure 1B). Argentation thin layer chromatography (Ag-TLC) analyses showed that like oleic acid, the unsaturated fatty acids (palmitoleic acid, *cis*-vaccenic acid, and linoleic acid) almost fully repressed *de novo* fatty acid biosynthesis (>95%). However, cells incubated with saturated fatty acids (palmitic acid or stearic acid) continued to synthesize fatty acids although palmitic acid had a moderate repression effect (5-fold decrease) whereas stearic acid failed to repress (Figure 1B). The *cis*-unsaturated fatty acids gave much stronger repression of fatty acid biosynthesis (100-fold decrease) than a *trans*-unsaturated fatty acid (5-fold decrease) or a cyclopropane fatty acid (10-fold decrease). Together these results indicated that exogenous *cis*-unsaturated fatty acids efficiently suppressed *E. faecalis de novo* fatty acid biosynthesis.

### FabT Mediates Regulation of *Enterococcus faecalis de novo* Fatty Acid Biosynthesis by Exogenous Fatty Acids

To test whether exogenous fatty acids regulate fatty acid synthesis through the FabT transcriptional repressor, *de novo* fatty acid biosynthesis of a *E. faecalis* Δ*fabT* strain was measured by [1-^14^C]acetate labeling in the presence of the individual fatty acids given above (Figures 1C,D). In the absence of FabT all exogenous fatty acid species including *cis*-unsaturated fatty acids failed to repress fatty acid biosynthesis. The one exception was palmitoleic acid, which gave a 20-fold decrease in fatty acid synthesis in the *E. faecalis* Δ*fabT* strain. However, palmitoleic acid strongly inhibited cell growth and is known to be a potent antimicrobial fatty acid (Parsons et al., 2012).

### Deletion of *fabT* Decreases *Enterococcus faecalis* Incorporation of Exogenous Fatty Acids

**FIGURE 2:**
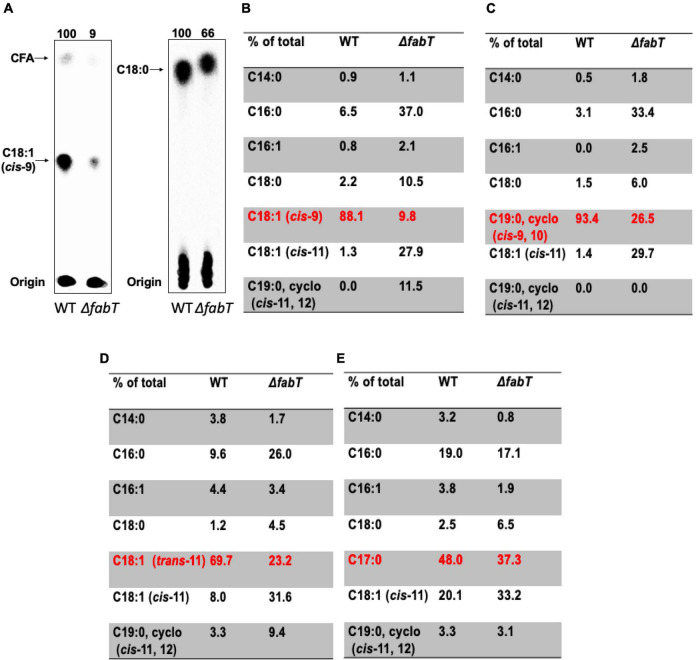
The *E. faecalis* Δ*fabT* strain is deficient in incorporation of exogenous fatty acids. **(A)** Incorporation of [^14^C]oleic acid (at left) or [^14^C]stearic acid (at right) into the cell membrane phospholipids of *E. faecalis* wild-type and Δ*fabT* strains. **(B)** The acyl chain composition of cell membrane phospholipids of *E. faecalis* wild-type and Δ*fabT* strains grown with oleic acid. **(C)** The acyl chain composition of cell membrane phospholipids of *E. faecalis* wild-type and Δ*fabT* strains grown with the cyclopropane fatty acid, methyleneoctadecanoic acid. **(D)** The acyl chain composition of cell membrane phospholipids of *E. faecalis* wild-type and Δ*fabT* strains grown with *trans*-vaccenic acid. **(E)** The acyl chain composition of cell membrane phospholipids of *E. faecalis* wild-type and Δ*fabT* strains grown with the C17 saturated acid, margaric acid. In panel **(A)**, the lane of Δ*acpB* (ZL318) cells at the right of the Δ*fabT* lane was deleted because of the missense mutation detected in its *fabT* gene (see below).

The regulatory role of FabT in fatty acid regulation of fatty acid synthesis was tested by the incorporation of several fatty acids in the wild type *E. faecalis* FA2-2 strain and the Δ*fabT* strain (Figure 2A). Incorporation of [1-^14^C]-labeled oleic acid or stearic acid by these two strains was assayed. The Δ*fabT* strain incorporated 10-fold less [1-^14^C]oleic acid that did the wild-type strain (Figure 2A) whereas [1-^14^C]stearic acid incorporation by the Δ*fabT* strain was only 34% less than the wild-type strain level (Figure 2A).

The effects of incorporation of exogenous fatty acids on phospholipid acyl chain compositions of *E. faecalis* wild-type and Δ*fabT* strains was also assayed by gas chromatography-mass spectrometry (GC-MS) analyses (Figures 2B–E). Relative to the wild type strain the Δ*fabT* strain showed much less incorporation of the unsaturated fatty acids (oleic acid, *trans*-vaccenic acid, or the cyclopropane methyleneoctadecanoic acid) into cell membrane phospholipids (Figures 2B–D) whereas incorporation of the 17-carbon saturated fatty acid (margaric acid) was very similar in the two strains (Figure 2E). The defective incorporation of unsaturated fatty acids by the Δ*fabT* strain seems likely to the result of increased synthesis of unsaturated fatty acids (Lane 1 of Figure 1C) which compete with the exogenous acids for incorporation into cell membrane phospholipid molecules, the quantity of which is limited by the size of the cell. This competition could act at several levels such as the PlsY and PlsC acyltransferases or the interconversion of acyl-phosphates and acyl-ACPs catalyzed by the freely reversible PlsX enzyme. Competition at both levels is also possible. Competition probably accounts for at least part the modest decreases in [1-^14^C]acetate incorporation into phospholipid acyl chains seen in the Δ*fabT* strain in the presence of *cis* unsaturated fatty acids, although it remains possible that an additional unknown weak regulatory process is involved.

### *Enterococcus faecalis* FabT Protein Is Functional *in vitro*

**FIGURE 3:**
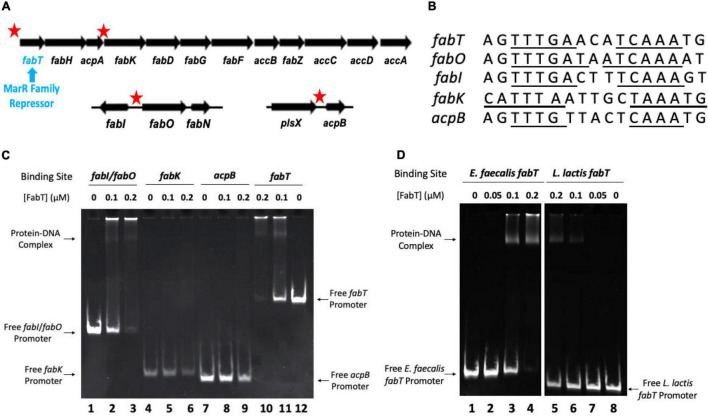
*Enterococcus faecalis* FabT protein is functional *in vitro*. **(A)** Four *E. faecalis fab*-related DNA gene fragments having putative FabT-binding sites were tested. The sequences tested are indicated by the red stars whereas the *fabT* gene is referred by the blue arrow. Note that the first fours genes were previously shown to be cotranscribed (Zhu et al., 2019) and a recent report showed that this is the case for the entire eleven gene operon (Hays et al., 2021). **(B)** The nucleotide sequences of FabT-binding sites of the promoters of *fab*-related genes above. The underlined sequences indicate the palindromes. **(C)** EMSA analysis of *E. faecalis* FabT binding to the *fabI*/*fabO* promoter, *fabK* promoter, *acpB* promoter, or *fabT* promoter. **(D)** EMSA analysis of *E. faecalis* FabT binding to the *E. faecalis fabT* promoter (at left) or *L. lactis fabT* promoter (at right). In panel **(D)**, both *fabT* promoters with 0.5 μM FabT lanes in the middle of *E. faecalis* group and *L. lactis* group were deleted since almost all *E. faecalis fabT* gene promoter fragments were trapped in the well and did not enter the gel.

In *E. faecalis* most of the fatty acid synthesis genes are transcribed as a single mRNA species encoding 12 genes (Zhu et al., 2019; Hays et al., 2021) (Figure 3A). The exceptions are the two genes (*fabO* and *fabN*) required for synthesis of the unsaturated acids and *fabI*, the gene encoding the essential enoyl-ACP reductase. Putative FabT binding sites were found all in *fab*-related genes. These sites were within the promoter regions of *fabT*, the *fabI*/*fabO* (enoyl-ACP reductase/β-ketoacyl-ACP synthase) operon, *fabK* (enoyl-ACP reductase), and *acpB* (Figures 3A,B). The *in vitro* binding ability of *E. faecalis* FabT protein to these promoters was tested using the electrophoretic mobility shift assay (EMSA). *E. faecalis* FabT bound the *fabT* and *fabI*/*fabON* promoter regions (Figure 3C) as expected from its regulatory role in fatty acid synthesis. However, FabT failed to bind hypothesized promoter regions of the *fabK* or *acpB* genes, neither of which play significant roles in *E. faecalis* fatty acid biosynthesis (Bi et al., 2014; Zhu et al., 2019). The *fabK* gene is coexpressed with *fabT*, *fabH*, and *acpA* genes encoded early in the *fab* gene operon (Figure 3A) but is inefficiently translated *in vivo* (Bi et al., 2014) whereas the role of the AcpB protein is incorporation of exogeneous fatty (Bi et al., 2014; Zhu et al., 2019), Note also that the *fabK* gene has been shown to be cotranscribed with the upstream and downstream genes (Zhu et al., 2019) and others have recently shown that all the genes of the FabT operon are cotranscribed (Hays et al., 2021). Hence, the *Streptococcus pneumoniae fabK* promoter (Jerga and Rock, 2009) is not present in *E. faecalis.*

*E. faecalis* FabT had no detectable binding affinity to the promoter region of *L. lactis f abT* (Figure 3D) indicating that FabT binding is selective and specific. These data demonstrate that purified *E. faecalis* FabT is functional *in vitro*.

### Exogenous Fatty Acids Enhance FabT Transcriptional Repression Activity

**FIGURE 4:**
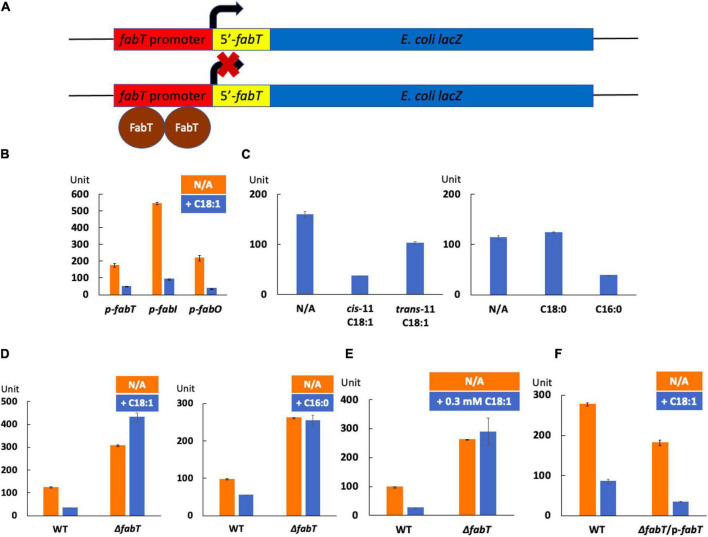
Exogenous fatty acids enhance transcriptional repression by *E. faecalis* FabT. **(A)** The β-galactosidase reporter plasmid constructs. **(B)** The effects of oleic acid on expression of β-galactosidase driven by the *E. faecalis fabT*, *fabI*, or *fabO* promoter. **(C)** The effects of exogenous unsaturated fatty acids (at left) or saturated fatty acids (at right) on production of β-galactosidase driven by the *E. faecalis fabT* promoter. **(D)** The effects of oleic acid (at left) or palmitic acid (at right) on production of β-galactosidase by the *fabT* promoter in the *E. faecalis* Δ*fabT* strain. **(E)** The effects of an increased concentration of oleic acid on production of β-galactosidase driven by the in *E. faecalis fabT* promoter in the Δ*fabT* strain. **(F)** The effects of oleic acid on expression of *lacZ* gene from *fabT* promoter in *E. faecalis* Δ*fabT*-complemented strain. The fatty acid concentration in the cultures was 0.1 mM for panels **(B–D,F**). In Panel **(E)** the concentration was 0.3 mM.

To test whether exogenous fatty acids regulate fatty acid synthesis by stimulating FabT binding, β-galactosidase reporter plasmids were constructed by fusing the promoters of the *E. faecalis fabT*, *fabI*, or *fabO* gene with the promoter-less *E. coli lacZ* gene of the pBHK322 plasmid constructed previously (Zhu et al., 2019) to give β-galactosidase expression regulated by FabT binding to its promoter (Figure 4A). These plasmids were transformed into the *E. faecalis* wild-type strain and β-galactosidase expression was assayed after incubation in the presence or absence of exogenous fatty acids. The presence of oleic acid led to a 3.5-fold decrease in β-galactosidase expression from the *fabT* promoter relative to the level seen in the absence of exogenous fatty acids (Figure 4B). A similar decrease in β-galactosidase expression was also detected for the *fabI* promoter (6-fold decrease) and the *fabO* promoter (6-fold decrease) in the presence of oleic acid (Figure 4B) indicating that exogeneous oleic acid stimulates FabT binding to these promoters and represses transcription of these *fab* genes. Note that FabT regulates its own expression as well as that of the 11 downstream genes.

We also tested β-galactosidase expression driven by the *fabT* promoter in the presence of various fatty acids. Both *cis*-vaccenic acid acid and palmitic acid repressed β-galactosidase expression (Figure 4C). However, *trans*-vaccenic acid reduced β-galactosidase expression by only 30% (Figure 4C) suggesting that this ligand is disfavored for FabT binding. In addition, stearic acid had no effect on β-galactosidase expression from the *fabT* promoter (Figure 4C). However, note that stearate is an only minor component of *E. faecalis* phospholipids.

The constructed reporter plasmids were transformed into the *E. faecalis* Δ*fabT* strain and the β-galactosidase expression from the *fabT* promoter was assayed (Figures 4D,E). The presence of oleic acid or palmitic acid failed to repress β-galactosidase expression in the Δ*fabT* strain (Figure 4D) and a 3-fold increase in concentration of oleic acid also had no effect (Figure 4E). However, repression was recovered when plasmid pQZ271 carrying the *E. faecalis fabT* gene was introduced (Figure 4F). These results demonstrate that exogenous fatty acids repress *E. faecalis de novo* fatty acid biosynthesis by stimulating FabT transcriptional repression.

### Acyl-AcpB Species Formed From Exogenous Fatty Acids Act as Ligands That Enhance FabT Repression

**FIGURE 5:**
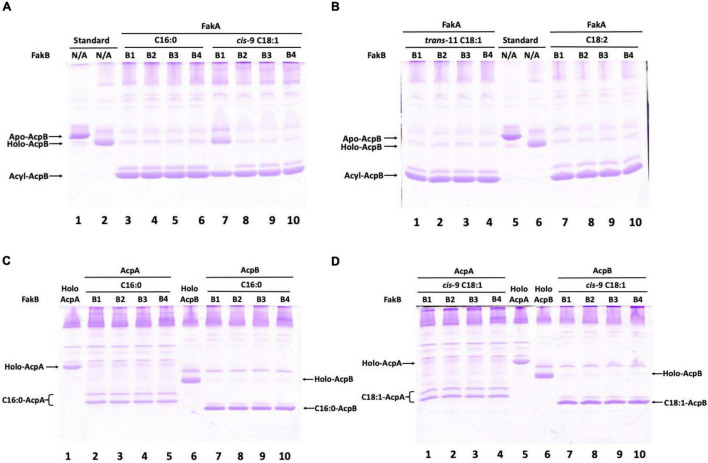
*Enterococcus faecalis* acyl-AcpB is formed by the Fak-PlsX system *in vitro*. **(A)** Synthesis of palmitoyl-AcpB and oleoyl-AcpB by the *E. faecalis* Fak-PlsX system. **(B)** Synthesis of *trans*-vaccenoyl-AcpB and linoleoyl-AcpB by the *E. faecalis* Fak-PlsX system. **(C)** Relative synthesis of palmitoyl-AcpA and palmitoyl-AcpB by the *E. faecalis* Fak-PlsX system. **(D)** Relative of synthesis of oleoyl-AcpA and oleoyl-AcpB by the *E. faecalis* Fak-PlsX system.

In *S. pneumoniae* FabT was reported to require fatty acyl-ACP for DNA binding (Zuo et al., 2019). In *E. faecalis* the two ACPs, AcpA and AcpB) are functionally expressed *in vivo* (Zhu et al., 2019). AcpA is thought to perform the essential carrier function in *de novo* fatty acid synthesis whereas AcpB functions in incorporation of exogenous fatty acids (Zhu et al., 2019). In the presence of exogenous oleic acid, *E. faecalis* represses expression of AcpA while AcpB expression continues (Bi et al., 2014; Zhu et al., 2019). This indicates that AcpB rather than AcpA regulates fatty acid synthesis by exogenous fatty acids accumulated by the fatty acid kinase components, FakA plus the four FakB proteins that bind the exogenous fatty acids for presentation to FakA. These proteins were purified as was the PlsX phosphate acyl transferase. FakA protein was incubated with one of the FakB binding proteins and then allowed to react with an *E. faecalis* holo-ACP (either AcpA or AcpB) in the presence of various fatty acids and PlsX (Figure 5). The results were analyzed on conformation-sensitive gels and showed that the *E. faecalis* Fak-PlsX reactions synthesized acyl-AcpB species from various fatty acids (Figures 5A,B). Notably, almost all of the holo-AcpB was converted to acyl-AcpB species whereas holo-AcpA was only partially converted to acyl-ACP species under the same conditions (Figures 5C,D). These data confirm that AcpB is preferred over AcpA in incorporation of exogenous fatty acids as previously observed in cell extracts (Zhu et al., 2019).

**FIGURE 6:**
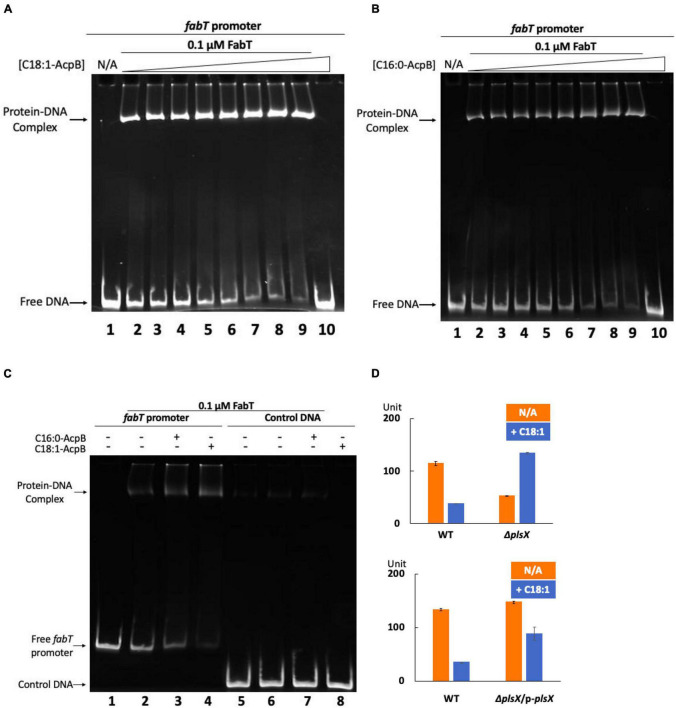
*Enterococcus faecalis* acyl-AcpB enhances FabT transcriptional repression. **(A)** EMSA analysis of *E. faecalis* FabT binding to the *fabT* promoter in the presence of increasing concentrations of oleoyl-AcpB. **(B)** EMSA analysis of *E. faecalis* FabT binding to *fabT* promoter in the presence of increasing concentrations of palmitoyl-AcpB. **(C)** EMSA analysis of *E. faecalis* FabT binding to the *fabT* promoter or to a control unrelated DNA fragment. **(D)** The effects of oleic acid supplementation on expression of β-galactosidase from the *fabT* promoter in *E. faecalis* Δ*plsX* (top panel) and Δ*plsX*-complemented (lower panel) strains.

To directly test whether the acyl-AcpB is the ligand that enhances *E. faecalis* FabT promoter binding, EMSA analysis of FabT binding to its predicted FabT promoter binding site (Figure 3A) was tested in the presence of acyl-AcpB (Figure 6). The results were analyzed using EMSA gels that showed that both oleyl-AcpB (Figure 6A) and to a lesser extent palmitoyl-AcpB (Figure 6B) enhanced binding to the *fabT* promoter indicating that acyl-AcpB enhances FabT transcription repression ability. In addition, the presence of palmitoyl-AcpB or oleoyl-AcpB failed to enhance *E. faecalis* FabT binding to a non-specific control DNA (Figure 6C).

### *Enterococcus faecalis* AcpB and FabT May Function in the Same Pathway *in vivo*

**FIGURE 7:**
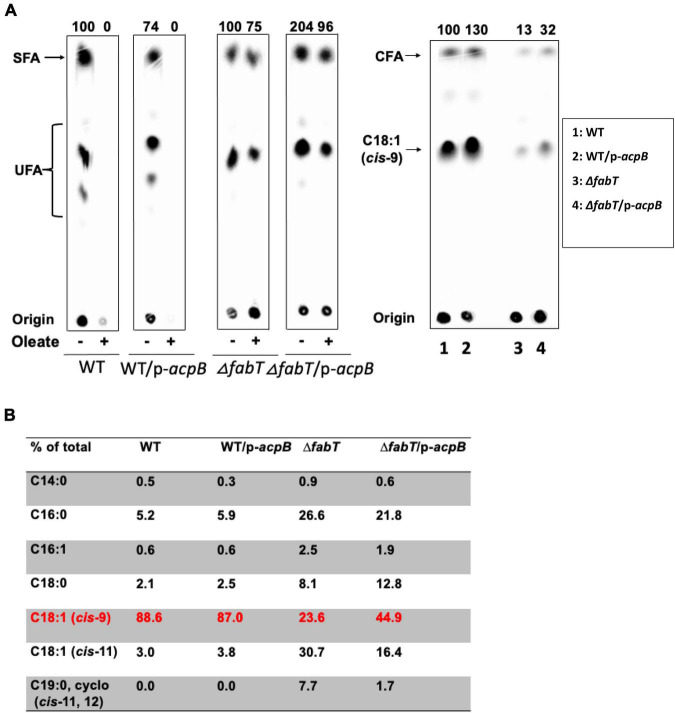
The regulatory function of *E. faecalis* AcpB for fatty acid biosynthesis relies on FabT. **(A)** *De novo* fatty acid biosynthesis of the *E. faecalis* wild-type strain overexpressing AcpB and Δ*fabT* strain overexpressing AcpB. Incorporation of [^14^C]oleic acid into the cell membrane phospholipids of the *E. faecalis* AcpB overexpression strain. **(B)** GC-MS analysis for proportion of oleic acid incorporation into the cell membrane phospholipids of the *E. faecalis* AcpB overexpression strain. For panels **(A)**, the lanes of cells with stearic acid at the right of each group were deleted since they were unrelated to this study. **(B)**, Two lanes for the Δ*acpB:cat* (ZL318) strain and its AcpB overexpression derivative at the right of Δ*fabT* group were deleted due to the FabT* mutation of this strain.

To study the interaction between *E. faecalis* FabT and AcpB *in vivo*, the Δ*plsX* strain which lacks the ability to synthesize acyl-AcpB ligands from exogenous fatty acids was transformed with the *lacZ* reporter plasmid to assay β-galactosidase expression from the *E. faecalis fabT* promoter in the presence of exogenous oleic acid (Figure 6D). The lack of PlsX decreased FabT-dependent β-galactosidase repression in the presence of oleic acid although this could be partially recovered by complementation of the Δ*plsX* strain with a plasmid-encoded *plsX* gene (Figure 6D). In addition, AcpB overexpression failed to overcome the inability of the Δ*fabT* strain to repress fatty acid synthesis in the presence of oleic acid (Figure 7A). Note that the Δ*fabT* strain showed increased fatty acid synthesis relative to the wild type strain in the absence of oleic acid. This seems likely to be due to FabT binding to its operators in the absence of its ligand (Figures 3C,D) as seen for many transcription factors (e.g., LacI). Moreover, the strain remained defective in incorporation of [1-^14^C]oleic acid (Figure 7A) and had a much lower level of oleic acid incorporation into cell membrane phospholipids relative to the wild-type strain (Figure 7B), although AcpB overexpression enhanced oleic acid incorporation relative to the Δ*fabT* strain (Figures 7A,B). These results seem likely to be due to increased competition for incorporation of exogenous versus *de novo* synthesized fatty acids into phospholipids. These data and those above suggest that FabT and AcpB function in the same pathway in exogenous fatty acid regulation of fatty acid synthesis. Interestingly, the Δ*plsX* strain had much lower β-galactosidase expression from the *fabT* promoter compared to the wild-type strain in the absence of exogenous fatty acid (Figure 6D). This seems to be due to accumulation of acyl-AcpA formed by fatty acid synthesis. In the absence of PlsX acyl-AcpA cannot be converted to the acyl-phosphate required for the PlsY-catalyzed primary acylation of *sn*-glycerol-3-phospate and accumulates due to the lack of the acyl acceptor required by PlsC. These data argue strongly that under these conditions acyl-AcpA can function as FabT binding ligand to stimulate transcriptional repression ability.

### *Enterococcus faecalis* Acyl-AcpA Weakly Enhances FabT Transcription Repression

**FIGURE 8:**
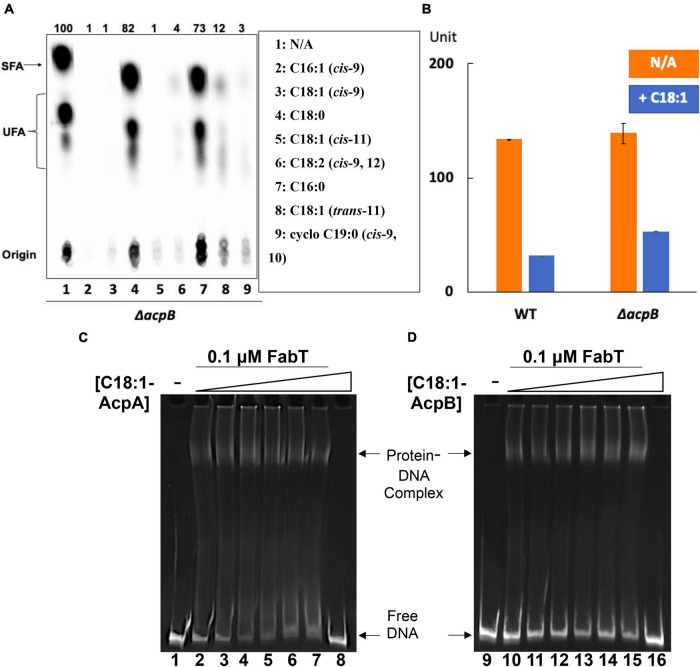
*Enterococcus faecalis* acyl-AcpA enhances FabT promoter binding in regulation of fatty acid synthesis. **(A)** *E. faecalis* Δ*acpB* strain *de novo* fatty acid synthesis in the presence of exogenous fatty acids. **(B)** The effects of oleic acid on expression of *lacZ* gene from *fabT* promoter in the *E. faecalis* Δ*acpB* strain. **(C,D)** EMSA analysis for comparison of *E. faecalis* FabT biding to the *fabT* promoter region in the presence of oleoyl-AcpA (at left) or oleoyl-AcpB (at right). The triangles denote increasing concentrations of acyl-ACP ligands These are (in μM): 0 for lanes 1, 2, 9, and 10; 0.25 for lanes 3 and 11; 0.5 for lanes 4 and 12; 1 for lanes 5 and 13; 2 for lanes 6 and 14; and 4 for lanes 7, 8, 15, and 16. In panels **(C,D)**, the binding of FabT to the *fabT* promoters in the presence of either of the two ligands were processed at the same time and the concentration of DNA promoters used for each situation was the same. But more smears for each lane could be detected in the oleoyl-AcpA lanes, which could lead to lower intensity of free DNA at the bottom. This might also be caused by the different buffers used for preparing oleoyl-AcpA and oleoyl-AcpB, respectively. The oleoyl-AcpB was synthesized and eluted in K-MES buffer (pH 6.1) whereas oleoyl-AcpA was synthesized and eluted in Tris-HCl buffer (pH 7.5) since AcpA precipitates in the acidic buffer used in all other EMSA analyses.

To test whether acyl-AcpA can act as a FabT enhancement ligand, the Δ*acpB* strain was tested for *de novo* fatty acid synthesis in the presence of various individual fatty acids by [1-^14^C]acetate labeling (Figure 8). Like the *E. faecalis* wild-type strain, unsaturated fatty acids showed significant repression of bacterial fatty acid biosynthesis whereas saturated fatty acids had almost no effect (Figure 8A), indicating that unsaturated acyl-AcpA species regulates fatty acid synthesis under these conditions. To further compare the regulatory roles of the two ACPs, the Δ*acpB* strain above was transformed with the reporter plasmid to test β-galactosidase expression from the *fabT* promoter in the presence of exogenous oleic acid (Figure 8B). The presence of oleic acid reduced β-galactosidase expression driven by the *fabT* promoter by 2.5-fold in the Δ*acpB* strain. These data argue that in *E. faecalis* AcpA can substitute for AcpB in FabT-mediated transcriptional regulation of *de novo* fatty acid biosynthesis. Another possibility is that in the presence of oleate most or all the AcpA become converted to oleoyl-AcpA and fatty acid synthesis becomes blocked due to a lack of AcpA. However, overproduction of AcpA from a strong plasmid promoter did not relieve repression by oleate and thus this second possibility can be eliminated (data not shown). To better study the regulatory function of AcpA in FabT-mediated regulation, *E. faecalis* FabT binding to *fabT* promoter in the presence of oleoyl-AcpA or oleoyl-AcpB was compared and analyzed by EMSA (Figures 8C,D). Compared with oleoyl-AcpB, oleoyl-AcpA could only enhance FabT binding to DNA fragment at low concentrations (0.25 and 0.5 μM, Lane 3 and 4 of Figure 8C) and this effect gradually disappeared with the increase of ligand concentration possibly due to aggregation. These data further indicates that oleoyl-AcpA is able to weakly enhance transcription repression activity of *E. faecalis* FabT in regulation of fatty acid biosynthesis.

## Discussion

Understanding the patterns of interplay among the two ACPs, PlsX and FabT is intrinsically complex due to several unusual features of the system. One is the autoregulatory nature of FabT expression which also affects AcpA expression. Accumulation of acyl-AcpA due to a lack of PlsX will bind FabT, thereby decreasing the expression of both FabT and AcpA (hence of acyl-AcpA). Another feature is that when PlsX is active and exogenous oleate is present, there is competition for incorporation into phospholipids, a sink of limited capacity. If an exogenously derived acyl chain is attached to a given phosphatic acid precursor molecule, then an endogenously-synthesized acyl chain is excluded (and vice versa). Finally, there are the different biases in how AcpA and AcpB function in the PlsX reaction. AcpA favors formation of acyl-phosphates whereas AcpB favors formation of acyl-ACPs. Both preferences make good physiological sense. In the absence of exogenous fatty acids roughly half of the acyl-AcpA must be converted to acyl-phosphate, the substrate of the PlsY catalyzed first step in phosphatidic acid synthesis, with the remaining acyl-AcpA used for the PlsC catalyzed second step (Figure 1). In contrast the Fak pathway used to incorporate exogenous fatty acids proceeds through acyl-phosphate so the PlsY substrate is not limiting and partial conversion to acyl-AcpB would ensure that both PlsY and PlsC have acyl donor substrates. In *S. pneumoniae* the three FakB proteins show specificity in fatty acid binding and presentation to the FakA kinase (Gullett et al., 2019). Although *E. faecalis* encodes four FakB proteins, the purified proteins show little or no ability to discriminate between the unsaturated oleic acid and the saturated palmitic acid (Figure 5). However, we do not know if all four *fakB* genes are expressed *in vivo* and the relative levels of expression. The only discrimination seen our *in vitro* data is in synthesis of acyl-AcpB from oleic acid which seems to interact poorly with FakB1. However, *in vivo* oleic acid is readily incorporated into phospholipids and allows growth of *E. faecalis* strains blocked in fatty acid synthesis when provided as the sole supplement. Hence the preference for exogenous *cis* unsaturated fatty acids over saturated fatty acids cannot be explained at the FakB level by our data and suggesting that fatty acid preference may be exerted at a later step perhaps by the PlsY-PlsC enzymes that acylate the phospholipid *sn*-glyerol-3-phosphate backbone.

FabT is required for repression of fatty acid synthesis in the presence of exogenous fatty acids with *cis* unsaturates being the most effective regulators (Figure 1). In the absence of FabT full transcription of the fatty acid synthesis genes results in high levels of acyl-AcpA species that outcompete exogeneous oleic acid for incorporation into phospholipids. In a Δ*fabT* strain incorporation of oleic acid is decreased almost 10-fold relative to the wild type strain. However, incorporation of the saturated acid, stearic acid, is decreased by only one-third. Plasmid-encoded *lacZ* fusions to the FabT promoter show that *fabT* transcription is autoregulatory as expected from the operon structure. Lack of FabT results in an ∼3-fold increase in β-galactosidase levels. Note that FabT regulation of its promoter appears somewhat weaker than FabT regulation of the *fabI* and *fabO* promoters.

In our previous report *acpB* was shown to be constitutively expressed even in the presence of oleate supplementation. As expected from those results FabT cannot bind the *acpB* promoter (Figure 3) and thus AcpB is ready to function when exogenous fatty acids become available. Here we report that acyl-AcpB species synthesized by Fak-PlsX system work as ligands to enhance FabT binding to promoters that contain the FabT binding site resulting in transcriptional repression of the *fab* genes. Note that neither AcpB overexpression nor a 3-fold increase in oleate concentration have any effect on the lack of regulation in the Δ*fabT* strain (Figures 1, 4, 7). Note that *Lactococcus lactis* IL1403, a close relative of *E. faecalis* that encodes a similar fatty acid synthesis operon plus FabT, PlsX and Fak proteins lacks AcpB. Despite this lack exposure of *L lactis* to exogenous fatty acids efficiently blocks fatty acid synthesis (Morgan-Kiss and Cronan, 2008; Eckhardt et al., 2013) consistent with acylated derivatives of the AcpA performing FabT regulation. There are bacteria in which AcpB is probably essential. For example, the *Lactobacillus johnsonii* genome is devoid of genes encoding fatty acid synthesis proteins except those encoding the AcpS *holo*-ACP synthase and an AcpB homolog that is 53% identical to *E. faecalis* AcpB encoded next to *plsX*. As the genome infers (Boekhorst et al., 2004), *L. johnsonii* requires fatty acid supplementation for growth and it seems likely that its AcpB functions in fatty acid uptake and glycerol 3-phosphate acylation.

Regulation of fatty acid synthesis by exogenous fatty acids has been studied by others in *S. pneumoniae* and in *E. faecalis* in this laboratory. Although both bacteria have FabT proteins and similar fatty acid synthesis operons regulated by FabT, the two bacteria have different unsaturated fatty acid synthesis mechanisms and the main enoyl-ACP reductase of *E. faecalis* is encoded outside the operon as are the genes for unsaturated fatty acid synthesis. The *S. pneumoniae* and *E. faecalis* FabT proteins are distinctly different. The two proteins are only 51% identical and the *S. pneumoniae* protein is considerably more basic (calculated pI of 9.15) than the *E. faecalis* protein (calculated pI of 7.94). A crystal structure of the *S. pneumoniae* FabT complexed with a promoter DNA sequence has been reported (Zhu et al., 2019). Unfortunately, the structure seems unreliable because of the 11 FabT interactions with the DNA reported there is only a single H-bond to a DNA base in each monomer (Zuo et al., 2019). The remaining interactions are with the sugar phosphate backbones of the two DNA strands and therefore lack sequence specificity. It is most difficult to view this model as a biologically relevant protein-DNA interaction, given the lack of discrimination needed for sequence specificity. The structure presented in that report seems to represent only a markedly basic protein binding to B-form DNA in a non-specific manner. Although, the EMSA data presented lack a control DNA for non-specific DNA binding, it is reported that in *S. pneumoniae* FabT binds only acyl-AcpB: acyl-AcpA cannot bind (Zhu et al., 2019). In contrast, acylated *E. faecalis* AcpA enhances FabT binding to the *fabT* promoter.

The PlsX phosphate acyltransferase plays a central role in modulating fatty acid synthesis in the presence of exogeneous fatty acids. Growth of a *E. faecalis* Δ*plsX* strain where *fab* genes are poorly expressed is restored by the addition of exogenous oleic acid. This phenotype seems likely to be caused by accumulation of *de novo* synthesized acyl-AcpA that functions as a binding ligand to enhance FabT *fab* gene repression. A supply of exogenous fatty acids would give synthesis of 1-acyl-sn-glycerol-3-phosphate for the PlsC acyltransferase and fatty acid synthesis would need to provide only acyl-AcpA for the PlsC acyltransferase. Consumption of acyl-AcpA by the PlsC acyltransferase would release FabT from the promoters of the biosynthesis genes to give an increased supply of acyl chains for PlsC. A puzzling result is that complementation with a plasmid borne wild type *plsX* gene only partially restored transcriptional repression of *fab* genes in the presence of oleic acid. High throughput genomic sequencing (Oxford Nanopore confirmed by Illumina sequencing) of the original Δ*plsX* strain identified an Asn9Ser missense mutation (A26 transition to G) in FabT. This mutation might affect the dimerization of FabT protein. Another possibility is that mixed dimers form that compromise wild type FabT activity. If these hypotheses posit lower promoter binding affinities would increase fatty acid synthesis and be an attempt of the bacterium to overcome the fatty acid auxotrophy caused by loss of *pls*X. The mutation is recessive to wild type FabT which fully restores oleic acid repression (data not shown).

**FIGURE 9:**
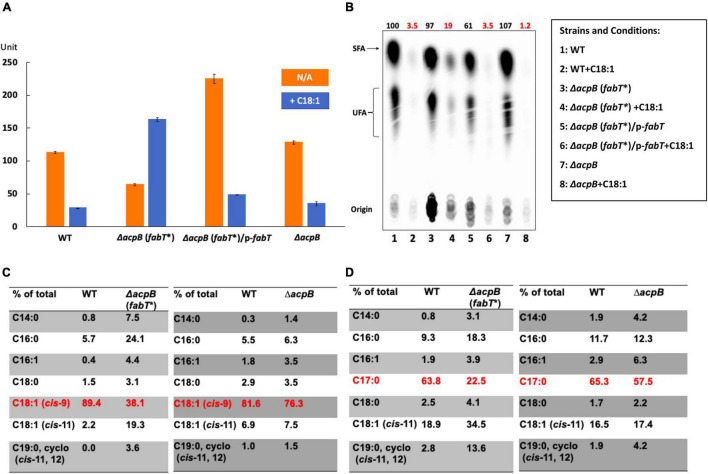
The FabT*mutation in the *acpB:cat* strain used previously results in a phenotype that differs from that of the newly constructed Δ*acpB* strain. **(A)** Comparison between the two Δ*acpB* strains of exogenous oleic acid supplementation on β-galactosidase production from the *fabT* promoter. The *pfabT* denotes a plasmid encoding wild type FabT. **(B)** Incorporation of [^14^C] acetate into cell membrane phospholipids of the two Δ*acpB* strains with additions as given. **(C)** The differing acyl chain compositions of the cell membrane phospholipids of the two Δ*acpB* strains grown in the presence of oleic acid Comparison of bacterial *de novo* fatty acid biosynthesis. **(D)** The differing acyl chain compositions of the cell membrane phospholipids of the two Δ*acpB* strains grown in the presence of margaric acid.

Reference to our prior report will show a conflict between the behavior of the Δ*acpB:cat* strain used in that work and the present Δ*acpB* strain. In the prior work addition of oleate to the Δ*acpB:cat* strain decreased fatty acid synthesis to about half that of the oleate-supplemented wild type strain. In the current work addition of oleate to the new Δ*acpB* strain decreased fatty acid synthesis down to a few percent of the level of fatty acid synthesis seen in parallel cultures of the strain that lacked oleate. Indeed, this phenotype is essentially identical to that of the wild type strain. The new Δ*acpB* strain was constructed because overexpression of AcpB in the Δ*acpB:cat* strain failed to fully restore transcriptional repression of *fab* genes in the presence of oleic acid. To address this conflict we performed whole genome sequencing as above and found that the Δ*acpB:cat* strain contains a mutation in *fabT* which we call fabT*. The fabT*mutation is Ala134Thr (G 400 transition to A) and FabT* has partial activity (Figure 9). In the presence of oleate FabT* represses fatty acid synthesis by about 5-fold rather than the >50-fold seen with wild type FabT. Modeling plus recourse to the *S. pneumoniae* FabT structure (Zuo et al., 2019) suggests that the hydrophobic residue to polar residue FabT* mutation might interrupt the hydrophobic tunnel that forms upon dimerization of FabT. The tunnel is proposed to bind the acyl-ACP acyl chain. If so, this might decrease binding of the FabT ligand and lead to high level expression in the presence of oleic acid. The fabT* phenotype is recessive to wild type *fabT* consistent with a loss of function mutation (Figure 9). However, the wild type FabT was produced in excess so FabT* could be outcompeted and mixed dimers of the wild type and mutant FabT monomers are another possibility. Since the Δ*acpB:cat* strain was constructed by homologous recombination and *fabT* and *acpB* are far apart on the genome it seems unlikely that the fabT* lesion was introduced in strain construction, but rather gave a selective advantage in the absence of AcpB. A plausible scenario is that the lack of AcpB compromises FabT regulation of fatty acid synthesis perhaps by altering the ratio of acyl-phosphate to acyl-AcpA and increased AcpA levels are needed to compensate thereby providing a selection for partial inactivation of FabT. The fact that *fabT* mutations arise in Δ*plsX* and Δ*acpB* strains which lack proteins that have no role in fatty acid synthesis demonstrates the strong coupling of FabT regulation to exogenous fatty acid utilization in *E. faecalis* phosphatic acid synthesis.

Our studies indicate the potential of acyl-AcpA as a binding ligand for enhancement of FabT binding. This differs from the *S. pneumoniae* report that only acyl-AcpB enhances FabT binding (Zuo et al., 2019) acyl-AcpA was reportedly inactive (Zuo et al., 2019). This seems surprising since *E. faecalis* and *S. pneumoniae* AcpAs are about 66% identical. However, this may partially explain the growth difference between *E. faecalis* Δ*plsX* strain and *S. pneumoniae* Δ*plsX* strain, in which the latter strain grows normally while the former one grows much more slowly relative to the wild-type strain. This also might be due to the less repression of *fab* gene expression by *S. pneumoniae* FabT since its binding may not be enhanced by *de novo* synthesized acyl-AcpA (Zuo et al., 2019). Experiments in which the *E. faecalis acpA* and *fabT* are replaced with the cognate *S. pneumoniae genes* may clarify the mechanisms that give what seem different AcpA-FabT interactions.

## Materials and Methods

### Materials

The *cis*-9, 10-ethyleneoctadecanoic acid was purchased from Cayman Chemicals and all of the other fatty acids, antibiotics, and ortho-nitrophenyl-β-galactoside (ONPG) were purchased from Sigma-Aldrich. DNA polymerases, restriction endonucleases, and T4 ligase were from NEB and the TA cloning kit was from Thermo-Fisher Scientific. Sodium [1-^14^C]acetate (specific activity, 58.6 mCi/mmol) and [1-^14^C]stearic acid (specific activity, 53.0 mCi/mmol) were provided by Moravek, Inc while the [1-^14^C]oleic acid (specific activity, 55 mCi/mmol) was purchased from American Radiolabeled Chemicals. Ni-NTA resin, 6% native PAGE gel and SYBR Green I nucleic acid stain were from Invitrogen and the DEAE anion-exchange column was from Sartorius. The DNA purification kits were from Qiagen, and the silver nitrate silica gel thin layer plates were from Analtech. All the other reagents were of the highest available quality. Oligonucleotide primers were synthesized by Integrated DNA Technologies and DNA sequencing was provided by ACGT Inc.

### Bacterial Strains, Plasmids, and Incubation

The bacterial strains and plasmids used in this study are listed in Supplementary Table 1 and the primers used for this study are listed in Supplementary Table 2. *E. coli* cells were incubated at 37°C in Luria-Bertani medium (tryptone, 10 g/L; yeast extract, 5 g/L; NaCl, 10 g/L) whereas *E. faecalis* cells were cultured at 37°C in AC medium (tryptone, 10 g/L; yeast extract, 10 g/L, K_2_HPO_4_, 5 g/L; glucose, 1 g/L). Antibiotics were added at the following concentrations (in mg/L): sodium ampicillin, 100 for *E. coli*; kanamycin sulfate 50 for *E. coli*; chloramphenicol 30 for *E. coli* and 10 for *E. faecalis*; and erythromycin at 250 for *E. coli* and 10 for *E. faecalis*. Fatty acids were added at 0.1 mM unless otherwise stipulated.

### Construction of *Enterococcus faecalis* Δ*fabT*, Δ*acpB*, and Δ*plsX* Strains

Strain constructions were performed as described previously (Blancato and Magni, 2010). To construct the Δ*plsX* strain the DNA cassette for the null Δ*plsX* gene was composed of a 500 bp upstream segment (arm I) and a 500 bp downstream segment (arm II) of the *plsX* gene coding region bracketing an *Xba*I site. The deletion cassette was constructed by overlap polymerase chain reaction (PCR) using primer sets EfplsX up *Nco*I F and EfplsX up *Xba*I R, and EfplsX down *Xba*I F, and EfplsX down *Pst*I R inserted into the temperature-sensitive *E. coli lacZ* gene vector pBVGh *Nco*I and *Pst*I sites. *E. faecalis* cells transformed with the constructed vector above were selected on AC agar plates with 10 mg/L erythromycin and 100 mg/L 5-bromo-4-chloro-3-indolyl-β-D-galactopyranoside (X-Gal) at 30°C. One blue colony was picked up and streaked on AC agar plates with the same components at 42°C to verify plasmid integration into the genome. The verified blue colony was cultured in AC liquid medium with 0.1 mM palmitate at 30°C for 4 h and then shifted to 42°C overnight. This process was repeated for several times and the culture was diluted and plated on AC agar plate containing 0.1 mM palmitate and 100 mg/L X-Gal at 42°C. White colonies were selected and validated for deletion of the target gene by colony PCR. The Δ*acpB* strain was constructed by the same procedures. The constructed deletion cassette obtained using primer sets EfacpB up *Sac*I Fand EfacpB up *Xba*I R, and EfacpB down *Xba*I F and EfacpB down *Pst*I R, was inserted into vector pBVGh at the *Sac*I and *Pst*I sites The final culture was diluted and plated on AC agar plate containing 100 mg/L X-Gal at 42°C and white colonies were selected and verified for target gene knockout through colony PCR.

### Construction of *Enterococcus faecalis acpB* and *plsX* Overexpression Plasmids

*Enterococcus faecalis acpB* and *plsX* genes were amplified from genomic DNA by PCR using primer set EfacpB *Nco*I F and EfacpB *Eco*RI R whereas the *plsX* gene was amplified using primer set EfplsX SamI F and EfplsX *Eco*RI R. For the *acpB* overexpression plasmid, the *acpB* fragment was digested with *Nco*I and *Eco*RI and ligated to the pZL277 vector cut with the same enzymes as in the previous work (Zhu et al., 2019). For the *plsX* overexpression plasmid, the acquired *plsX* fragment was digested *Sma*I and *Eco*RI and ligated with pZL277 vector cut with the same enzymes. The resulting plasmid was further processed by Quick-Change PCR at the *Nco*I site to obtain the correct open reading frame.

### Purification of *Enterococcus faecalis* FabT, FakA, FakB, and PlsX Proteins

The vector for *E. faecalis* FabT protein expression in *E. coli* was constructed by inserting the *fabT* coding fragment amplified using primer set EffabT *Nde*I F and EffabT *Eco*RI R into the vector pET28b *Nde*I site and *Eco*RI sites. The expression vector for *E. faecalis* PlsX proteins was constructed by inserting its coding fragment of each gene into pET28b vector at the *Nde*I site and *Hin*dIII sites. The expression vectors for *E. faecalis* FakA and FakB proteins were constructed by inserting the coding fragment of each gene amplified by primer sets EffakA *Nco*I F and EffakA *Eco*RI R, EffakB1 *Nco*I F and EffakB1 *Eco*RI R, EffakB2 *Nco*I F and EffakB2 *Eco*RI R, EffakB3 *Nco*I F and EffakB3 *Eco*RI R, and EffakB4 *Nco*I F and EffakB4 *Eco*RI R, respectively into the *Nco*I and *Eco*RI sites of pET28M vector, which was modified from pET28b vector by exchanging the positions of *Nco*I and *Nde*I on the plasmid.

To express the proteins above, the *E. coli* Rosetta transformed with the expression vectors above were cultured at 37°C to OD_600_ of 0.6 and then induced with 1 mM isopropyl β-D-1-thiogalactopyranoside (IPTG) for 4 h. The cells were harvested, washed by phosphate-buffered saline, resuspended with lysis buffer containing 50 mM Na-PO_4_ (pH 7.0) for FabT while pH 8.0 for the other proteins, 0.3 M NaCl, 10 mM imidazole, and 1 mM DTT and then lysed with a French Pressure Cell Press. The supernatant was loaded onto Ni-NTA columns, and the target proteins were eluted with 0.25 M imidazole, dialyzed and stored with 20% glycerol at −80°C. An SDS-gel of the purified PlsX FabT and the five Fak proteins is given as Supplementary Figure 1. Figure 5 gives the purified AcpA and AcpB protein in the standards lanes.

### Synthesis of *Enterococcus faecalis* Acyl-ACPs

*Enterococcus faecalis* AcpA and AcpB were converted into functional holo-AcpA and holo-AcpB using the methods described in the previous work (Zhu et al., 2019). To synthesize *E. faecalis* acyl-ACP species, holo-AcpA and holo-AcpB were mixed with various 0.2 mM free fatty acids irrespectively in the presence of *E. faecalis* 1 μM FakA, 1 μM FakB, and 0.5 μM PlsX proteins in buffer containing 50 mM Tris-HCl (pH 7.5), 2 mM MgCl_2_, 1 mM DTT, and 0.2 mM ATP and then incubated at 37°C for 45 min. The products were analyzed on 2M urea-18% PAGE conformation-sensitive gel electrophoresis.

To obtain acyl-ACPs for electrophoretic mobility shift assays (EMSA), the reactions above were incubated for 3 h and the products were further purified through VIVAPURE D Mini H columns, eluted with 0.5 M LiCl or NaCl, and stored at −80°C with 10% glycerol.

### Thin Layer Chromatography Analysis of Radioactive Labeled Fatty Acid Methyl Esters From Membrane Phospholipids

To test bacterial *de novo* fatty acid biosynthesis, *E. faecalis* strains were started at OD = 0.1 in AC medium and incubated at 37°C in the presence of 1 mCi/L sodium [1-^14^C]acetate with or without single exogenous fatty acid at 0.1 mM. The cells were lysed by methanol-chloroform (2:1) solution and the phospholipids were extracted in chloroform and then dried under nitrogen. The fatty acyl groups were methylated by 25% (w/v) sodium methoxide, extracted by hexanes, and processed for TLC analysis on Analtech silica gel containing 20% silver nitrate in toluene at −20°C. The plates containing the [^14^C] labeled fatty acid methyl esters were exposed on the phosphorimager GE Typhoon FLA700 Scanner) and then analyzed by ImageQuant TL software.

Monounsaturated methyl esters separate from saturated esters because the double bonds interact with the silver nitrate in the plate (π-π interactions). Under the right conditions (toluene as solvent run at −20°C), monounsaturated methyl esters separate according to the position of the double bond. Hence, Δ9 C16 runs slower than Δ11 C18 and Δ9 C16 and Δ9 C18 run together.

To test bacterial incorporation of exogenous unsaturated fatty acids, *E. faecalis* strains were started at OD of 0.1 in AC medium and incubated at 37°C to OD_600_ values of about 1.6 ± 10%, in the presence of 0.1 mCi/L [1-^14^C] oleic acid with 0.1 mM non-radioactive oleate. The cells were washed with phosphate-buffered saline and the phospholipids were extracted, methylated, and processed for TLC analysis as described above. To test bacterial incorporation of exogenous saturated fatty acids, *E. faecalis* cultures were started at OD of 0.1 in AC medium and incubated at 37°C to OD_600_ values of about 1.6 ± 10% in the presence of 0.1 mCi/L [1-^14^C]stearic acid with 0.1 mM non-radioactive palmitate and processed for TLC analysis as described above.

### Gas Chromatography-Mass Spectrometry Analysis of Incorporated Fatty Acids of Cell Membrane Phospholipids

*Enterococcus faecalis* strains were inoculated at an OD of 0.1 in AC fatty acid free medium and incubated with 0.1 mM oleic acid, *trans*-vaccenic acid, methyleneoctadecanoic acid, or margaric acid at 37°C. The cells were lysed by methanol-chloroform (2:1) solution and the phospholipids were extracted into chloroform and dried by nitrogen. The fatty acyl groups were methylated by 25% (w/v) sodium methoxide in methanol, extracted with hexanes and sent for gas chromatography-mass spectrometry analysis.

### Electrophoretic Mobility Shift Assay

The *E. faecalis fabT* promoter of about 400 bp was obtained by PCR using primer set EffabT promoter F and EffabT promoter R and 10 nM of the DNA fragment was incubated with 0.1 μM FabT protein and 0∼10 μM acyl-ACP in the binding buffer containing 10 mM Tris-HCl (pH 7.0), 50 mM NaCl, 1 mM EDTA, 1 mM DTT, and 10% glycerol at room temperature for 30 min. The mixture was separated by 6% native PAGE gel, stained with SYBR Green I Nucleic Acid Gel Stain, and analyzed through Quantity One software.

### β-Galactosidase Assays

To construct the β-galactosidase reporter plasmid, the promoter and the first 35 bp of the *E. faecalis fabT* coding sequence (−389 to +35 relative to *fabT* gene initiation codon ATG) was amplified by PCR using primer set EffabT promoter plus 35 *Pst*I F and EffabT promoter plus 35 *Sal*I R and inserted into the *Pst*I and *Sal*I sites at the 5′ end of the promoterless *lacZ* gene on plasmid pBHK322 constructed in the previous work (Bi et al., 2014). The vector expressing β-galactosidase from the *E. faecalis fabI* or *fabF2* promoter was constructed as above using primer sets EffabI promoter plus 35 *Pst*I F and EffabI promoter plus 35 *Sal*I R, and EffabO promoter plus 35 *Pst*I F and EffabO promoter plus 35 *Sal*I R, respectively. To coexpress the *lacZ* and *fabT* genes, the PCR-amplified *fabT* fragment plus the p32 promoter using primer set p32 *Sma*I F and EffabT *Sma*I R from plasmid pZL278 as template was digested with *Sma*I and inserted into the *Sma*I site located downstream of *lacZ* gene on the constructed reporter vector.

For β-galactosidase assays *E. faecalis* strains containing the constructed *lacZ*-expression vectors above were cultured at 37°C to mid-log phase, harvested by centrifugation, washed with phosphate-buffered saline, resuspended in Z buffer, lysed with chloroform and sodium dodecyl sulfate, and assayed for β-galactosidase activity at 30°C. The data were obtained in triplicate from at least two independent experiments.

## Data Availability Statement

The datasets presented in this study can be found in online repositories. The names of the repository/repositories and accession number(s) can be found below: NCBI GenBank – CP085841.

## Author Contributions

QZ and HD designed and performed experiments. LZ provided materials. JC designed experiment and wrote the manscript with QZ. All authors contributed to the article and approved the submitted version.

## Conflict of Interest

The authors declare that the research was conducted in the absence of any commercial or financial relationships that could be construed as a potential conflict of interest.

## Publisher’s Note

All claims expressed in this article are solely those of the authors and do not necessarily represent those of their affiliated organizations, or those of the publisher, the editors and the reviewers. Any product that may be evaluated in this article, or claim that may be made by its manufacturer, is not guaranteed or endorsed by the publisher.
