# Supplementary material for: The Enterococcus faecalis FabT Transcription Factor Regulates Fatty Acid Biosynthesis in Response to Exogeneous Fatty Acids
Source: Front Microbiol. 2022 Apr 25;13:877582. doi: 10.3389/fmicb.2022.877582 (PMC9083066; doi:10.3389/fmicb.2022.877582)
Supplement: Supplementary file 1 [file Data_Sheet_1.docx]

Supplementary Materials

The *Enterococcus faecalis* FabT Transcription Factor Regulates Fatty Acid Biosynthesis in Response to Exogeneous Fatty acids.

Qi Zou, Huijuan Dong, Lei Zhu and John E. Cronan

**Table S1 Strains and Plasmids**

| **Strains and Plasmids** | **Description** | **Source** |
| --- | --- | --- |
| **Strains** |  |  |
| *E. coli* Rossetta | *ompT hsdSB* (rB^-^ mB^-^) *gal dcm* (DE3) pRARE (Cm^r^) | Novagen |
| *E. faecalis* FA2-2 | Wild Type | Lab Store |
| *E. faecalis* ZL116 | *∆fabT* | Zhu et al., 2019 |
| *E.faecalis* ZL318 | *∆acpB::cat* with Cm^r^ in genome | Zhu et al., 2019 |
| *E. faecalis* DHJ523 | *∆acpB* | This work |
| *E. faecalis* QZ157 | *∆plsX* | This work |
| *E. faecalis* QZ12 | *∆fabT* with *acpB* expression plasmid | This work |
| *E. faecalis* QZ14 | FA2-2 with *acpB* expression plasmid | This work |
| *E. faecalis* QZ219 | FA2-2 with *lacZ* expression vector from *fabT* promoter | This work |
| *E. faecalis* QZ239 | FA2-2 with *lacZ* expression plasmid from *fabI* promoter | This work |
| *E. faecalis* QZ241 | FA2-2 with *lacZ* expression plasmid from *fabO* promoter | This work |
| *E. faecalis* QZ223 | *∆plsX* with *lacZ* expression plasmid from *fabT* promoter | This work |
| *E. faecalis* QZ261 | *∆acpA* with *lacZ* expression plasmid from *fabT* promoter | This work |
| *E. faecalis* QZ277 | *∆acpB* with *lacZ* expression plasmid from *fabT* promoter | This work |
| *E. faecalis* QZ167 | *∆plsX* with *plsX* expression plasmid | This work |
| *E. faecalis* QZ290 | *∆plsX* with *plsX* expression plasmid and *lacZ* expression plasmid from *fabT* promoter | This work |
| *E. faecalis* QZ251 | *∆fabT* with *lacZ* expression plasmid from *fabT* prmoter | This work |
| *E. faecalis* QZ274 | *∆fabT* with *lacZ-fabT* co-expression plasmid | This work |
| *E. faecalis* QZ55 | *∆acpB::cat* | This work |
| *E. faecalis* QZ272 | *∆acpB::cat* with *lacZ*-*fabT* co-expression plasmid | This work |
|  |  |  |
| **Plasmids** |  |  |
| pBVGh | Temperature-sensitive β-galactosidase erythromycin-resistant gene modification plasmid | Blancato et al., 2010 |
| pZL277 | Shuttle plasmid with a p32 promoter, *E. faecalis* expression | Zhu et al., 2019 |
| pZL391 | *E. faecalis plsX* expression plasmid | Zhu et al., 2019 |
| pZL167 | *E. faecalis acpB* expression plasmid | Zhu et al., 2019 |
| pZL130 | *E. faecalis fabT* expression plasmid | Zhu et al., 2019 |
| pZL484 | pET28M plasmid modified from pET28b by exchanging the NcoI site and NdeI sites | This work |
| pQZ123 | *E. faecalis fakA* expression plasmid | This work |
| pZL490 | *E. faecalis fakB1* expression plasmid | This work |
| pQZ126 | *E. faecalis fakB2* expression plasmid | This work |
| pQZ120 | *E. faecalis fakB3* expression plasmid | This work |
| pZL488 | *E. faecalis fakB4* expression plasmid | This work |
| pZL278 | *E. faecalis fabT* in pZL277 | Zhu et al., 2019 |
| pQZ13 | *E. faecalis acpB* in pZL277 | This work |
| pQZ114 | *E. faecalis plsX* in pZL277 | This work |
| pZL274 | *E. faecalis acpB* knockout cassette in vector pBVGh | This work |
| pQZ149 | *E. faecalis plsX* knockout cassette in vector pBVGh | This work |
| pBHK322 | promoterless *E. coli* *lacZ* in vector pTRKL2 | Bi et al., 2014 |
| pQZ214 | *E. faecalis fabT* start region (-389 to +35) at 5'-end of *lacZ* in pBHK322 | This work |
| pQZ235 | *E. faecalis fabI* start region (-297 to +35) at 5'-end of *lacZ* in pBHK322 | This work |
| pQZ238 | *E. faecalis fabO* start region (-297 to +35) at 5'-end of *lacZ* in pBHK322 | This work |
| pQZ271 | p32 promoter with *E. faecalis fabT* downstream of *lacZ* in pQZ214 | This work |

**Table S2 Oligonucleotides primers used in the study**

| **Primers*** | **Sequence 5'-3'** |
| --- | --- |
| EfacpB up SacI F | ATTAAGAGCTCTAGCTGACGTGGTGGTGACAG |
| EfacpB up XbaI R | CTTATTATTTTTTATCTAGACAACTGTATTCACCTCCACTG |
| EfacpB down XbaI F | ACAGTTGTCTAGATAAAAAATAATAAGACAAAGTCG |
| EfacpB down PstI R | AAAATTCTGCAGGTAATTTTGGTATGATTGGG |
| Cm XbaI F | GAATTCCATATGTCTAGAGTCGACGGCAATAGTTAC |
| Cm XbaI R | AAGCTTGTCGACTCTAGACATATGATCCTGGAGCTGTAAT |
| EffabT NdeI F | GCATCATATGGTGCACAGAATGGAACC |
| EffabT EcoRI R | GATCGAATTCTTATTTGTATTCTTGCAAGAAATC |
| EfplsX NdeI F | CTAGCATATGAAAATTGCTGTAGATGC |
| EfplsX HindIII R | ATGCAAGCTTACTCTGCTTTGCCTTCATA |
| pET28M F | ATCACAGCAGCGGCCTGGTGCCGCGCGGCAGCCCCATGGCTAGCATGACTGGTG |
| pET28M R | GCGGCACCAGGCCGCTGCTGTGATGATGATGATGATGGCTGCTGCCCATATGATATCTCC |
| EffakA NcoI F | GTCACCATGGTGAATGTAACAGAAATCAGC |
| EffakA EcoRI R | GTACGAATTCTTATTCTGCTGAGAATAAGTATG |
| EffakB1 NcoI F | CTAGCCATGGTGAAAATTGCTATTGTGACAG |
| EffakB1 EcoRI R | ATGCGAATTCTTATTGAGCTGAAATCGCTAAAC |
| EffakB2 NcoI F | CAGTCCATGGTGACAAACGTTAAAATCGTAAC |
| EffakB2 EcoRI R | GACTGAATTCTTAGTCTGTATAGTACATAATAG |
| EffakB3 NcoI F | CTAGCCATGGTGAATAAAGAAAAAATCGCAC |
| EffakB3 EcoRI R | GTACGAATTCTTAATCATTTAATAACTGTATG |
| EffakB4 NcoI F | CATGCCATGGTGAATTATCAATTAGTGACAG |
| EffakB4 EcoRI R | GACTGAATTCTTATTTTCTTTTTTCACCAAAAG |
| EffabK promoter R | GTCAGGATCCGTCGACATTGATTTTATACGTCCTTTAG |
| EffabK promoter F | GTACGAATTCTGCAGTAATACATGAAAATAGGGGG |
| EffabT promoter R | GTCAGGATCCGTCGACATCCATATACTTTGATTATC |
| EffabT promoter F | GTCAGAATTCTGCAGTAATTAATCGTCCTACATTTTA |
| EfacpB promoter F | GTCAGAATTCTGCAGTAATTTGCAATTGTCTTGAC |
| EfacpB promoter R | GTCAGGATCCGTCGACATCTGTATTCACCTCCACTGC |
| LlfabT promoter F | GCCTAATGAATTCAAAAGTTACT |
| LlfabT promoter R | TTTCATCTGTCATCCTCTTCATT |
| EffabO promoter R | GTCAGGATCCGTCGACATACTGTACCTCCGAGTTTA |
| EffabO promoter F | GTCAGAATTCTGCAGCATATAAAATCTCCTTTAATA |
| EfacpB NcoI F | CATGCCATGGCTTTGACTCGTGAAGAAGTA |
| EfacpB EcoRI R | CCGGAATTCTTATGAATTACTCACAATATAATCT |
| EfplsX up NcoI F | CATGCCATGGCACAGTTACATCAGTTACG |
| EfplsX up XbaI R | TTCATAATATTCCACTCTAGATCCACCCATTGCATCTA |
| EfplsX down XbaI F | GATGCAATGGGTGGATCTAGAGTGGAATATTATGAAGGCAAA |
| EfplsX down PstI R | AAAACTGCAGTGTAAAAATAGCCTTATTGCTTAATAAAAT |
| EfplsX SmaI F | TCCCCCGGGATGAAAATTGCTGTAGATGC |
| EfplsX EcoRI R | CCGGAATTCTTACTCTGCTTTGCC |
| pZL277-EfplsX NcoI QC F | GAGGTGAACCATGCCCG |
| pZL277-EfplsX NcoI QC R | CGGGCATGGTTCACCTC |
| EffabT promoter plus 35 PstI F | AAAATTCTGCAGTTAATCGTCTTACATTTTAATAAG |
| EffabT promoter plus 35 SalI R | ACGCGTCGACAAGTAATCGTTGACTGT |
| EffabI promoter plus 35 PstI F | AAAATTCTGCAGACTGTACCTCCGAGTTTATTT |
| EffabI promoter plus 35 SalI R | ACGCGTCGACCCCATTACGACGACATT |
| EffabO promoter plus 35 PstI F | AAAATTCTGCAGATAAAATCTCCTTTAATAAAAAATTTTTGTTGATAGAATAGGCTT |
| EffabO promoter plus 35 SalI R | ACGCGTCGACACGGCACCCATGCCG |
| p32 SmaI F | TCCCCCGGGAGATTAATAGTTTTAGCTA |
| EffabT SmaI R | TCCCCCGGGTTATTTGTATTCTTGCAAGA |

***** The primer sequences were designed using the *E. faecalis* V583 genome. The underlined sequences indicate the restriction sites used in the study.

**Supplementary Figure S1**


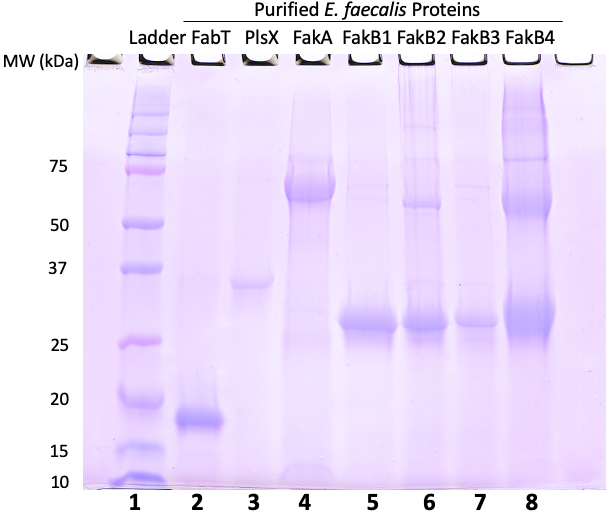


Figure S1: SDS-PAGE gel electrophoretic analysis of purified proteins. There are *E. faecalis* FabT (~18 kDa, Lane 2), PlsX (~36 kDa, Lane 3), FakA (~60 kDa, Lane 4), FakB1 (~30 kDa, Lane 5), FakB2 (~30k Da, Lane 6), FakB3 (~30k Da, Lane 7) and FakB4 (~30 kDa, Lane 8),
